# Identification of Non-HIV Immunogens That Bind to Germline b12 Predecessors and Prime for Elicitation of Cross-clade Neutralizing HIV-1 Antibodies

**DOI:** 10.1371/journal.pone.0126428

**Published:** 2015-05-26

**Authors:** Zheng Yang, Jingjing Li, Qingsheng Liu, Tingting Yuan, Yanyu Zhang, Li-Qing Chen, Qi Lou, Zehua Sun, Huazhong Ying, Jianqing Xu, Dimiter S. Dimitrov, Mei-Yun Zhang

**Affiliations:** 1 AIDS Institute, Department of Microbiology, Li Ka Shing Faculty of Medicine, The University of Hong Kong, Pok Fu Lam, Hong Kong, China; 2 Center of Laboratory Animals, Zhejiang Academy of Medical Sciences, Zhejiang, China; 3 Institutes of Biomedical Sciences, Shanghai Public Health Clinical Center, Fudan University, Shanghai, 201508, China; 4 Protein Interactions group, Laboratory of Immunology, Cancer and Inflammation Program, Center for Cancer Research, National Cancer Institute, National Institutes of Health, Frederick, Maryland, United States of America; 5 Liver Disease Institute, Shenzhen Third People’s Hospital, Shenzhen, 518112, China; Shanghai Medical College, Fudan University, CHINA

## Abstract

A fundamental challenge for developing an effective and safe HIV-1 vaccine is to identify vaccine immunogens that can initiate and maintain immune responses leading to elicitation of broadly neutralizing HIV-1 antibodies (bnAbs) through complex maturation pathways. We have previously found that HIV-1 envelope glycoproteins (Env) lack measurable binding to putative germline predecessors of known bnAbs and proposed to search for non-HIV immunogens that could initiate their somatic maturation. Using bnAb b12 as a model bnAb and yeast display technology, we isolated five (poly)peptides from plant leaves, insects, *E*. *coli* strains, and sea water microbes that bind to b12 putative germline and intermediate antibodies. Rabbit immunization with the (poly)peptides alone induced high titers of cross-reactive antibodies that neutralized HIV-1 isolates SF162 and JRFL. Priming rabbits with the (poly)peptides followed by boosts with trimeric gp140_SF162_ and then resurfaced Env (RSC3) induced antibodies that competed with mature b12 and neutralized tier 1 and 2 viruses from clade B, C and E, while control rabbits without (poly)peptide priming induced antibodies that did not compete with mature b12 and neutralized fewer isolates. The degree of competition with mature b12 for binding to gp140_SF162_ correlated with the neutralizing activity of the rabbit IgG. Reversing the order of the two boosting immunogens significantly affected the binding profile and neutralization potency of the rabbit IgG. Our study is the first to provide evidence that appears to support the concept that non-HIV immunogens may initiate immune responses leading to elicitation of cross-clade neutralizing antibodies.

## INTRODUCTION

The ability to elicit broadly neutralizing antibodies (bnAbs) is a holy grail for the development of an effective and safe HIV-1 vaccine. Many new bnAbs identified in recent years are more potent than the four well-known bnAbs b12 [1], 2G12, 2F5 and 4E10; however, those bnAbs were isolated from limited number of HIV-1-infected “elite controllers” [2–8]. Some of the newly identified bnAbs recognize the CD4 binding site (CD4bs) or have epitopes that overlap with the CD4bs on gp120, including HJ16 [8], VRC01-03 [4], VRC-PG04, 05, VRC-CHs [5], and NIH45-46, 8ANCs, 3BNCs and 12A21 [9]. Many other bnAbs recognize conformational epitopes that may involve loops on gp120 and require a glycan as part of their epitopes or linear epitopes in the membrane proximal external region (MPER) on gp41. Some bnAbs have been crystallized and their neutralizing epitopes identified [9–16]. The design of vaccine immunogens has focused on the neutralizing epitopes of known bnAbs, including 2F5, b12, 2G12 and VRC01. Various approaches have been used to design immunogens that target the epitopes of these bnAbs, including the use of linear or constrained peptides containing the 2F5 epitope or scaffolds presenting 2F5 binding determinants [17, 18], glycan-masking of non-neutralizing epitopes that do not affect b12 epitope [19–21], expression of non-glycosylated outer domain-derived gp120 fragments bearing the b12 epitope [22, 23], construction of fully cleavable Env trimer [24], and engineering of outer domain of gp120 to present VRC01 epitope [25]. Although these approaches, all of which are based on the HIV-1 Env, have not been successful in eliciting the same or similar bnAbs, some have generated cross-clade neutralizing HIV-1 antibodies (nAbs) with limited breadth [23, 24].

We and others have reported that bnAbs are highly divergent from their putative germline Abs, and the germline Abs of known bnAbs lack measurable binding to wild-type HIV-1 Env [3, 4, 25–28], indicating that somatic maturation of the germline predecessor antibodies of HIV-1 bnAbs may not be initiated by HIV-1 infection or vaccination with Env. This finding may partially explain why immunogens designed to include the neutralizing determinants of some known bnAbs have failed to elicit the same or similar bnAbs. Putative VRC01 germline IgG1 antibody has been reported to possess mM affinity for Env [5, 15]; however, a minimum affinity of μM (10^–6^ M) is typically required to trigger somatic hypermutation of naïve B-cells, or they cannot compete with other B-cells in the germinal center for clonal expansion [29, 30]. Based on these observations, we hypothesized that somatic maturation of HIV-1 bnAbs could be also initiated by exposure of the host to non-HIV primary immunogens, leading to the generation of intermediate antibodies (iAbs) that can bind Env and quickly mature to bnAbs following HIV-1 infection or vaccination with Envs (secondary immunogens) [31–33]. Such iAbs may exist in some HIV-1 uninfected human individuals due to pre-exposure to the primary immunogen(s), which enables the immune system to rapidly respond to HIV-1 infection and effectively contain the virus. Using CD4bs bnAb b12 as a model antibody, we isolated a panel of b12 iAbs from HIV-1 uninfected human individuals and rhesus macaques [33]. Intermediate Abs to bnAb CH103 were also detected in HIV-1 uninfected individuals [34]. Furthermore, by deep sequencing a large non-immune human IgM antibody library, we demonstrated the potential presence of iAbs to other HIV-1 bnAbs in healthy humans [35]. In the present study, we isolated five non-HIV (poly)peptides, dubbed P1-4 and P6, that bind to human and macaque b12 putative germline Abs and iAbs from recombinant yeast libraries constructed using genomic DNA fragments from various sources. We tested the isolated (poly)peptides in rabbits, alone or in combination with gp140_SF162_ trimer and a resurfaced Env, RSC3, for their ability to initiate and guide the immune responses towards b12-like bnAbs. RSC3 was designed based on HXB2 core for better binding to CD4bs bnAbs [4, 15]. We included RSC3 in the rabbit immunization with an attempt to focus the immune responses on CD4bs. We found that non-HIV (poly)peptides alone induced cross-reactive nAbs, and priming rabbits with the (poly)peptides followed by boosts with Envs enhanced elicitation of cross-clade nAbs.

## MATERIALS AND METHODS

### Cell lines, plasmids, peptides, Envs and antibodies

ZM-bl was obtained from the NIH AIDS Research and Reference Program (ARRP) (Division of AIDS, NIAID). HIV-1 Env plasmids and consensus clade B 15-mer peptides were obtained from the NIH ARRP or kindly provided by Linqi Zhang (Tsinghua University) [36]. Recombinant plasmids encoding RSC3 and gp140_SF162_ trimer were kindly provided by Peter Kwong and John Mascola (Vaccine Research Center, NIAID). Recombinant RSC3 and gp140_SF162_ trimer were produced in our laboratory using a 293F transient transfection system (Invitrogen) and Immobilized Metal Affinity Chromatography (IMAC). Putative human and rhesus macaque b12 germline single-chain antibody fragments (scFvs), previously identified five human b12 scFv iAbs and 10 rhesus macaque b12 scFv iAbs [33], and mature IgG1 b12 were produced in our laboratory. The following reagents were purchased: penicillin/streptomycin (Sigma), horse radish peroxidase (HRP)-conjugated goat anti-rabbit IgG Fc and phycoerythrin (PE)-conjugated goat anti-human IgG (Fab’)_2_ (Jackson ImmunoResearch), PE-conjugated streptavidin and FITC-conjugated goat anti-c-myc mouse IgG (Sigma).

### Preparation of genomic DNA fragments from various sources and construction of recombinant yeast display libraries

Genomic DNA from plant leaves, insects, *E*. *coli* strains, and sea water microbes were extracted, fragmented and cloned to yeast display vector pYD7 according to a previously described cloning strategy [37]. Vitis Vinifera, Eutrema Salsugineum, Oryza Sativa and Nicotiana Tabacum leaves were mixed equally by fresh weight, and genomic DNA was extracted using the cetyltrimethyl ammonium bromide (CTAB) method [38]. Insect genomic DNA was also prepared using the CTAB method. Bacterial cells from three *E*. *coli* strains (TG1, HB2151 and XL1-blue) were collected and equally mixed. Sea water microbes were prepared by centrifugation of 5 L of sea water at 10,800 x g for 15 min. Genomic DNA of bacteria and sea water microbes was extracted using Wilson’s bacteria genomic DNA extraction protocol [39]. Each 2 μg of genomic DNA was digested with 0.9 U of DNase I (Roche) at 15°C for 15 min in a total volume of 50 μL of digestion buffer (50 mM Tris-HCl, pH 7.5, 10 mM MnCl2). Multiple digestions were conducted to scale up fragmentation of the genomic DNA. Reactions were stopped by addition of EDTA to a final concentration of 50 mM followed by flash freezing in liquid nitrogen and incubation at 90°C for 10 min to inactivate the DNase I. Randomly digested genomic DNA was analyzed by 2% agarose gel electrophoresis, and fragments ranging from 100 bp to 500 bp were extracted from the gel. Purified fragments were blunt-ended using T4 DNA polymerase (New England Biolabs) and ligated to a modified pComb3X vector, pComb3X-SmaI, linearized with Sma I [37]. Large amount of recombinant plasmid DNA were prepared using Maxi Kit (Qiagen) and the inserts were PCR-amplified using three pairs of primers as previously described [37]. The PCR products were gel-extracted and re-amplified by PCR using a primer pair designed to add overhangs homologous to pYD7 [37]. The re-amplified inserts were mixed with linearized PYD7 plasmid DNA at a ratio of 3:1 (w/w) and electroporated into competent yeast cells, EBY100 [40], resulting in three recombinant yeast display libraries (plant + insects, *E*. *coli*, and sea water microbe). Each library contained over 10 million individual recombinant yeast clones.

### Sorting of yeast display libraries and screening for monoclonal yeast

Induction and sorting of yeast libraries and screening for monoclonal yeast were carried out according to a previously described protocol [37, 41]. Over 100 million induced yeast cells obtained from equal mixture of three yeast libraries were used in the 1^st^ round of sorting against 500 nM biotinylated human or macaque b12 germline scFv (200 nM and 50 nM for the 2^nd^ and 3^rd^ rounds of sorting, respectively). The 4^th^, 5^th^ and 6^th^ rounds of sorting were carried out following incubation with 500, 200 and 50 nM biotinylated human or macaque b12 iAbs, respectively. The sorted libraries were screened for monoclonal yeast clones that bind to human and macaque b12 germline and iAbs by flow cytometry. Plasmids were extracted from positive yeast clones using a yeast cell plasmid extraction kit (Omega Bio-Tek), amplified in *E*. *coli* strain TG1, and then sent for DNA sequencing. The inserted sequences were analyzed by using BLAST-N and BLAST-P.

### Rabbit immunization and sample collection

This study was approved by the HKU Committee on Using Live Animals in Teaching and Research (CULATR # 2297–10). The isolated (poly)peptides were synthesized (Genscript and American Peptides) and conjugated through the N-terminus to keyhole limpet haemocyanin (KLH) and/or expressed as Fc fusion proteins. A total of 10 female New Zealand White (NZW) rabbits were separated into five groups (two rabbits per group) and immunized with isolated (poly)peptides alone or boosted with gp140_SF162_ trimer and / or RSC3 (Table 1) using an extended 87-day protocol [42]. A total of 250 μg of (poly)peptides P1-4 mixed with Freund’s complete adjuvant (FCA) were used for the primary intradermal inoculation. Rabbits were then boosted subcutaneously with 125 μg of (poly)peptides mixed with Freund’s incomplete adjuvant (FIA) two weeks after the primary inoculation, and the boosts were repeated five more times at two- or three-week interval (Table 1). Five or ten milliliters of whole blood samples were collected prior to the primary inoculation and 10 days after the 2^nd^, 4^th^ and 6^th^ boosts. The collected samples were designated bleed 0, 2, 4 and 6, respectively. Rabbit IgGs were purified from heat-inactivated sera using protein G affinity purification. Rabbits were sacrificed at the end of the study; 133 days post the primary inoculation. Prior to the sacrifice, rabbits were intramuscularly injected with xylazine hydrochloride, an anesthetic, at a dose of 0.2 mL / kg body weight. No rabbit exhibited obvious pain or distress during the experiment.

**Table 1:** Protocol for rabbit immunization.

Injection	Day	Group 1	Group 3	Group 4	Group 5	Group 6
Primary (intradermal)	0	P1-4	gp140	RSC3	P1-4	P1-4
Boost 1 (s.c.)	14	P1-4	gp140	RSC3	P1-4	P1-4
Boost 2 (s.c.)*	35	P1-4 + gp140	gp140	RSC3	P1-4 + RSC3	P1-4
Boost 3 (s.c.)	49	gp140	gp140	RSC3	RSC3	P1-4
Boost 4 (s.c.)	70	gp140	gp140	RSC3	RSC3	P1-4
Boost 5 (s.c.)	87	RSC3	RSC3	gp140	gp140	P1-4
Boost 6 (s.c.)	108	RSC3	RSC3	gp140	gp140	P1-4

Notes:

* Equal amounts of (poly)peptides and recombinant Env (gp140_SF162_ trimer or RSC3) were mixed.

P1-4: Equal amounts of synthesized P1-, P2-, P3- and P4-KLH conjugates were mixed.

### Binding assays

Indirect ELISA was used for titration of rabbit sera or determination of binding activity of purified serum IgGs as follow: Microwell plates were coated with 2 μg/mL of gp140_SF162_ trimer or RSC3, or 5 μg/mL of each (poly)peptide, blocked with 2.5% skim milk in PBS (MPBS), and then three- or five-fold serially diluted rabbit sera or IgGs were added and incubated at 37°C for 1 h. Bound rabbit IgGs were detected using HRP-conjugated goat anti-rabbit Fc as a secondary antibody and TMB as a substrate. The optical density (OD) at 450 nm was measured after color development at RT for 20 min. EC50, EC20 and EC10 (50%, 20% and 10% maximum binding, respectively) were determined using GraphPad Prism software. The serum dilution at which OD450nm = EC10 was set as the serum titer. Competition ELISA was performed similarly, except that a fixed concentration of biotinylated IgG1 b12 leading to 70% maximum binding was added simultaneously to the wells, and bound IgG1 b12 detected using streptavidin-HRP as a secondary antibody (1:3,000) and ABTS as a substrate. Non-biotinylated IgG1 b12 was used as a positive control. The percent competition inhibition at 100 μg/mL of rabbit IgGs was equal to [1-(OD_100μg/ml_-OD_blank_)/(OD_max_-OD_blank_)].

### Neutralization assay

A standardized TZM-bl cell line-based Env-pseudotyped neutralization assay was used as previously described [43].

### Statistical analysis

Data from six experimental groups were combined, and the mean differences between two groups were compared by one-way ANOVA using SPSS software. P-values were determined.

## RESULTS

### Identification of (poly)peptides binding to human and macaque b12 predecessor germline antibodies and iAbs

**Fig 1:**
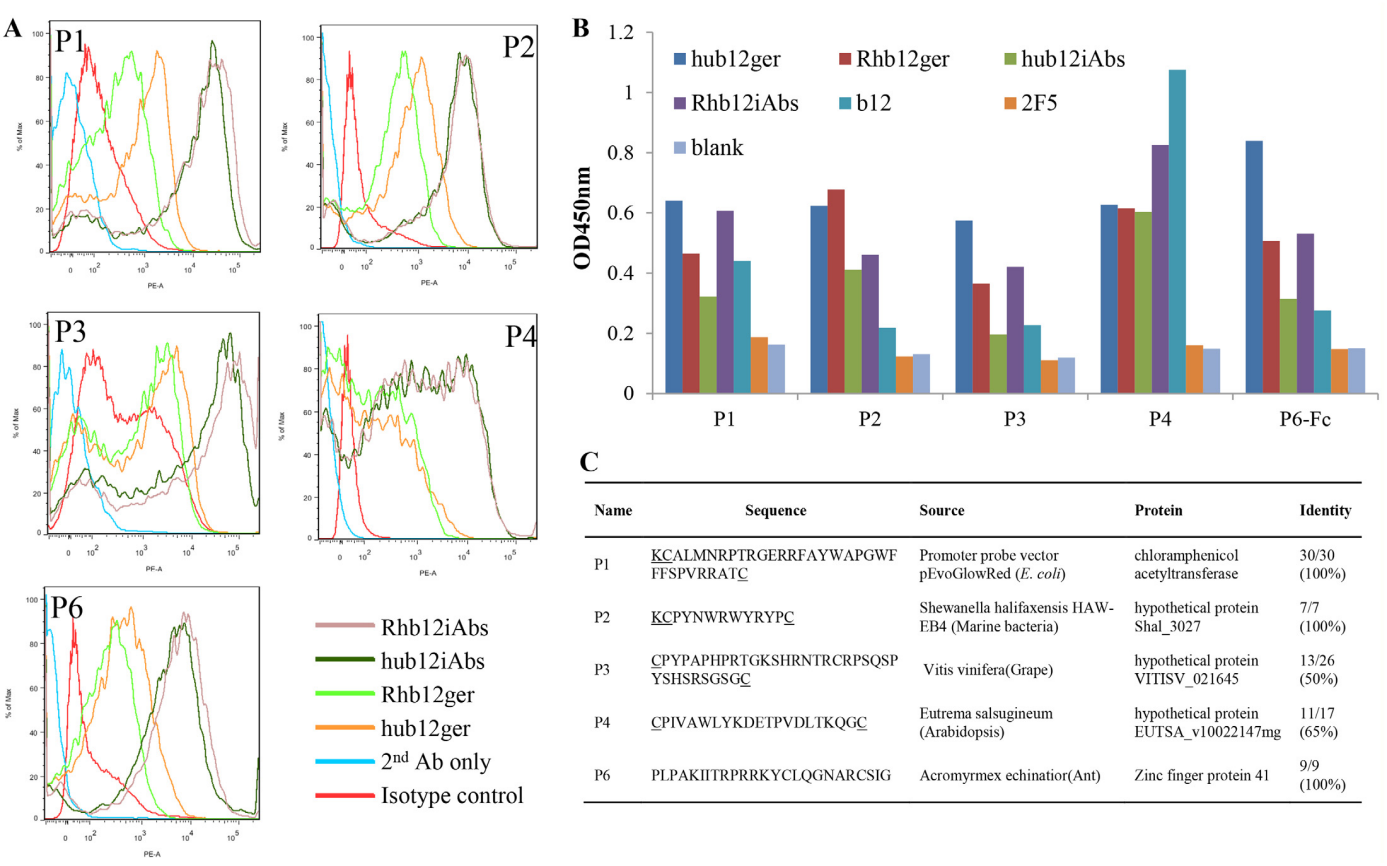
Identification of five (poly)peptides that bind to b12 germline and iAbs. A: Flow cytometry of yeast-displayed P1-4 and P6 stained with biotinylated human (hub12ger) and macaque (Rhb12ger) b12 germline scFvs, and human (hub12iAbs) and macaque (Rhb12iAbs) b12 iAbs. Isotype control: human mAb unrelated to Env gp120. B: Binding of human and macaque b12 germline scFvs, iAbs and mature IgG1 b12 at 50 μg/ml to the isolated (poly)peptides by ELISA. IgG1s 2F5 and VRC01 were included as controls. The blank control contained no primary antibody. C: Sequences and origins of the isolated (poly)peptides. Underlined amino acids were added when constrained (poly)peptides were synthesized as conjugates to KLH.

We constructed three recombinant yeast libraries using genomic DNA prepared from plants and insects, *E*. *coli* strains, and sea water microbes, respectively. Following six rounds of sorting against human or macaque b12 germline scFv or iAbs, over 200 monoclonal yeast clones were screened, and all of the clones that were positive for human and macaque b12 germline scFv and iAbs were sequenced. Five unique clones, P1-4 and P6, were isolated (Fig 1A) and their capacity to specifically bind b12 germline scFvs and iAbs confirmed by ELISA using synthesized constrained (poly)peptides P1-4 and recombinant P6-Fc fusion (Fig 1B). Interestingly, P1 and P4 also bound well to mature b12. Sequence analysis revealed that the isolated (poly)peptides were derived from different species and lacked sequence similarities to Envs (Fig 1C).

### Immunization with non-HIV (poly)peptides alone induced high titers of cross-reactive antibodies

**Fig 2:**
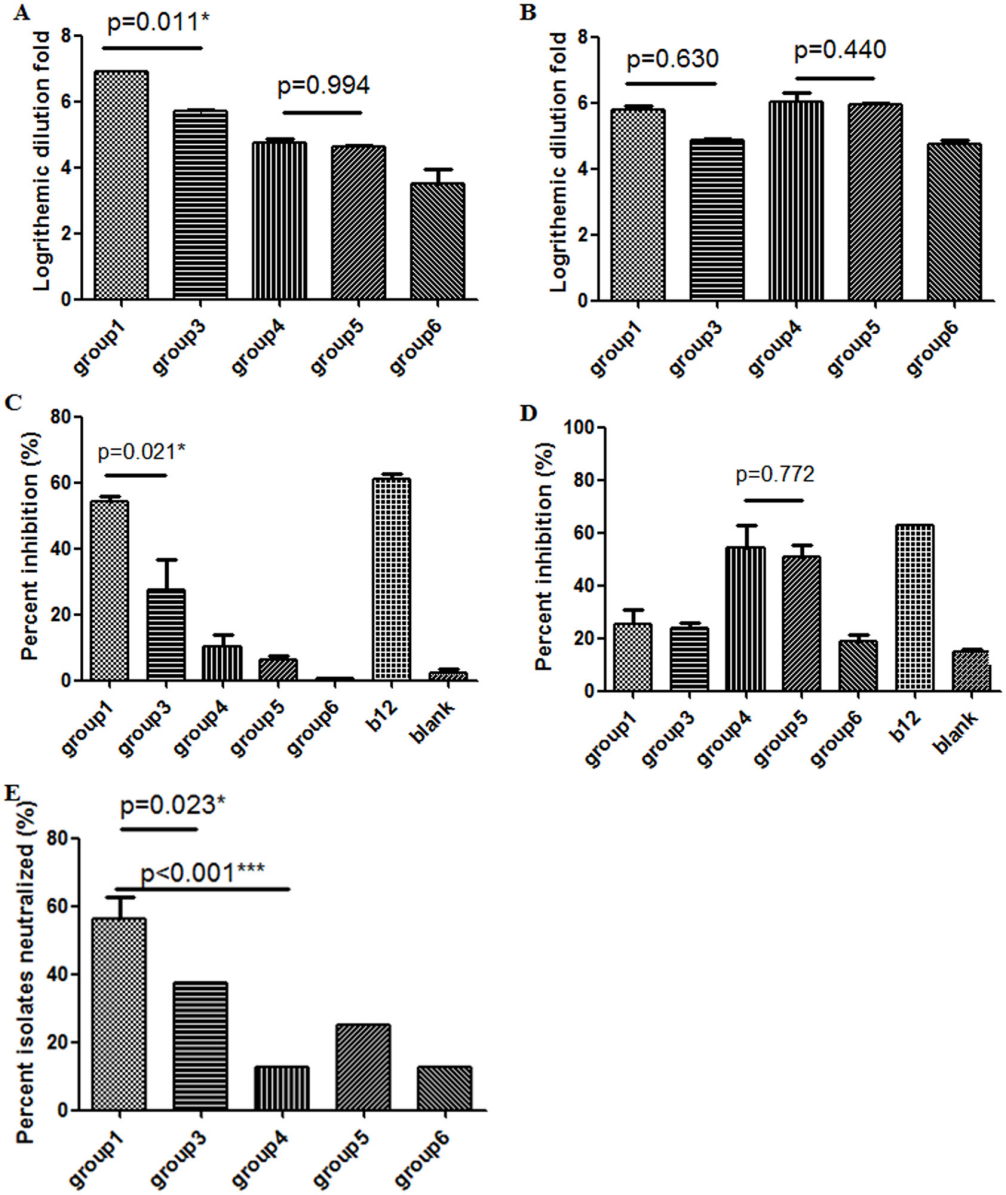
Characterization of bleed 6 sera and IgGs for binding and neutralization activities. A-B: Titration of bleed 6 sera for gp140_SF162_ trimer (A) and RSC3 (B). C-D: Competition of rabbit IgGs with mature human IgG1 b12 for binding to gp140_SF162_ (C) and RSC3 (D). Mature IgG1 b12 was included as control. For “blank”, no IgG was added. E: Neutralization breadth. Percent isolates neutralized by rabbit IgGs from each group (IC_50_ below 150 μg/mL) is shown. IgG1 b12 was tested at a maximum concentration of 20 μg/ml in the TZM-bl assay. IC_50_ > 20 μg/mL was defined as non-neutralizing. One-way ANOVA was used for statistical analyses using SPSS. Pre-immunization rabbit IgGs from each rabbit were also tested and all IC_50s_ were > 150 μg/mL (not shown).

Among the five groups of rabbits, group 6 was immunized with the synthesized (poly)peptides P1-4 alone (Table 1). Group 6 IgGs showed very low EC50s for the (poly)peptides (Table 2), indicating that these (poly)peptides were immunogenic. Importantly, high titers of serum antibodies to the gp140_SF162_ trimer and RSC3 were present in group 6 rabbits (Fig 2A and 2B) although group 6 IgGs were unable to compete with mature b12 for binding to gp140_SF162_ trimer or RSC3 (Fig 2C and 2D). Furthermore, group 6 IgGs neutralized two clade B isolates, SF162 and JRFL, albeit with relatively high IC50s (Table 3), which suggested that these (poly)peptides might structurally mimic HIV-1 Env. This notion was supported by the finding that two control groups (groups 3 and 4) without (poly)peptide priming produced cross-reactive, albeit to less extent, antibodies with P1-4 (Tables 1 and 2).

**Table 2:** Binding of bleed 4 IgGs to the isolated (poly)peptides by ELISA.

EC50 (μg/mL)	Group 1	Group 3	Group 4	Group 5	Group 6
P1	3.30 ± 2.68	> 150	> 150	0.89 ± 0.76	2.77 ± 3.00
P2	1.61 ± 0.52	> 150	> 150	2.62 ± 3.56	2.65 ± 3.37
P3	1.88 ± 0.03	> 150	> 150	1.01 ± 0.88	2.92 ± 2.90
P4	< 0.07	43.25 ± 4.33	83.1 ± 8.31	< 0.07	< 0.07
EC20 (μg/mL)	Group 1	Group 3	Group 4	Group 5	Group 6
P1	0.42 ± 0.23	50.4 ± 2.50	37.00 ± 3.70	0.12 ± 0.04	0.15 ± 0.06
P2	0.61 ± 0.59	68 ± 3.40	47.45 ± 3.46	< 0.07	0.07 ± 0.01
P3	0.71 ± 0.18	69.3 ± 1.98	50.2 ± 2.50	0.17 ± 0.08	0.28 ± 0.18
P4	< 0.07	38 ± 3.80	11.02 ± 5.78	< 0.07	< 0.07

Notes: EC50 and EC20 are antibody concentrations leading to 50% and 20% maximum binding, respectively. Pre-immunization rabbit IgGs from each rabbit were also tested and all EC_50s_ were > 150 μg/mL (not shown).

**Table 3:** Neutralization activities (IC50s) of the immune rabbit IgGs in the TZM-bl assay.

Tier	Subtype	HIV-1 isolate	Group 1		Group 3		Group 4		Group 5		Group 6		b12
1A	B	SF162	1.42±0.14	1.39±0.14	1.93±0.10	0.66±0.03	3.87±0.38	29.30±1.17	25.8±3.10	1.83±0.31	-	130.00±36.4	0.71±0.10
1B	B	HXB2	150.00±27.00	142.5±5.7	115.72±4.63	58.08±1.74	-	-	-	-	-	-	0.01±0.00
		Bal	109.00±4.36	-	-	-	-	-	-	-	-	-	0.15±0.02
	C	ZM109F.PB4	-	-	-	-	-	-	-	-	-	-	-
2	A	92UG037.8	-	-	-	-	-	-	-	-	-	-	-
	B	JRFL	63.90±0.64	106.00±1.06	70.00±3.50	68.4±2.05	-	-	-	150±4.50	88±7.92	-	0.06±0.00
		JRCSF	-	134.90±8.09	-	-	-	-	-	-	-	-	0.06±0.00
		89.6	-	-	-	-	-	-	-	-	-	-	0.01±0.00
		92HT594	-	-	-	-	-	-	-	-	-	-	3.41±0.34
		WITO4160.33	-	-	-	-	-	-	-	-	-	-	17.04±1.20
		SC422611.8	-	-	-	-	-	-	-	-	-	-	6.21±0.62
		CAAN5342	-	-	-	-	-	-	-	-	-	-	-
	C	GX-C44	-	-	-	-	-	-	-	-	-	-	14.47±0.72
		DU172.17	-	-	-	-	-	-	-	-	-	-	1.22±0.12
		93MW959	69.86±3.50	24.1±1.20	21.27±1.05	11.71±0.56	115.24±2.40	-	-	-	-	-	0.02±0.00
		ZM233M.PB	-	-	-	-	-	-	-	-	-	-	-
	07_BC'	CH110.2	-	-	-	-	-	-	-	-	-	-	-
	B'C	PCNE15	-	-	-	-	-	-	-	-	-	-	6.70±0.70
	01_AE	PCNE3	-	-	-	-	-	-	-	-	-	-	-
	D	Z2Z6	-	-	-	-	-	-	-	-	-	-	19.50±2.90
	E	GX-E14	125.30±3.76	-	-	-	-	-	122.78±12.30	-	-	-	17.5±1.75
3	B	TRJO4551.58	-	-	-	-	-	-	-	-	-	-	-
	08_BC	PCNE30	-	-	-	-	-	-	-	-	-	-	17.22±1.70
		PCNE50	-	-	-	-	-	-	-	-	-	-	-
	07_BC'	CH115.12	-	-	-	-	-	-	-	-	-	-	-
		CH120.6	-	-	-	-	-	-	-	-	-	-	-
**Accumulated percent isolates neutralized (%)**			**26.9**		**15.4**		**7.7**		**11.5**		**7.7**		**53.8**

All rabbit IgGs were tested at the highest concentration of 150 μg/mL followed by three-fold serial dilutions in duplicate or in triplicate. “-”: IC50 > 150 μg/mL. IgG1 b12 was tested at a maximum concentration of 20 μg/ml. IC50 > 20 μg/mL was defined as non-neutralizing. VSV were included in each neutralization assay as a negative control virus and the IC50s for all samples were > 150 μg/mL (not shown). Pre-immunization rabbit IgGs from each rabbit were also tested and all IC_50s_ were > 150 μg/mL (not shown).

### Priming rabbits with (poly)peptides followed by boosts with gp140_SF162_ and then RSC3 induced antibodies that competed with mature b12 and neutralized cross-clade isolates

To test whether the (poly)peptides could serve as primary immunogens, we primed rabbits with synthesized P1-4 (group 1 and 5) and boosted with gp140_SF162_ trimer and then RSC3 (group 1), or RSC3 and then gp140_SF162_ trimer (group 5). Group 3 and 4 serve as respective control groups without (poly)peptide priming (Table 1). Recombinant gp140_SF162_ trimer and RSC3 used for immunization were pre-tested by ELISA with a panel of Env-specific mAbs, including CD4bs mAbs VRC01, b12, m14 and m18, CD4-induced mAb X5, and gp41-specific mAb m44, and the quality of both proteins was confirmed (S1 Fig). Bleed 0, 2, 4 and 6 sera were titrated against gp140_SF162_ and RSC3. High titers of sera for both Env proteins were induced in all of the rabbits (S2 Fig). We compared bleed 6 serum titers for gp140_SF162_ and RSC3. Group 1 sera showed significantly higher titers for gp140_SF162_ trimer than control group 3 (Fig 2A). Alteration of the order of the two boosting immunogens in group 5 rabbits resulted in 100-fold lower serum titers for gp140_SF162_ (Fig 2A and S2A Fig). No significant difference in serum titer for RSC3 was observed between (poly)peptide priming groups and the corresponding control groups (Fig 2B and S2B Fig). We then measured b12-competing antibodies in bleed 6 IgGs. Group 1 IgGs significantly inhibited the binding of mature b12 to gp140_SF162_ trimer compared to the control group 3 IgGs (Fig 2C). However, group 5 IgGs did not compete with mature b12 for binding to gp140_SF162_ trimer (Fig 2C). Surprisingly, there was no significant difference between (poly)peptide priming groups and the corresponding control groups without priming in the degree of competition of rabbit IgGs with mature b12 for binding to RSC3 (Fig 2D). The degree of competition with mature b12 for binding to the gp140_SF162_ trimer seems to correlate with the neutralization activity of rabbit IgGs (Fig 2C–2E). Group 1 IgGs neutralized all three tier 1 clade B isolates tested and four tier 2 clade B (JRFL and JRCSF), C (93MW959) and E (GX-E14) isolates, while the control group 3 IgGs neutralized only two of the tier 1 and two of the tier 2 isolates neutralized by group 1 IgGs (Table 3). There was no significant difference between group 5 and 4 IgGs in neutralizing this panel of isolates tested (Fig 2E).

### The order of boosting immunogens significantly affected the antibody profile of immunized rabbits

**Fig 3:**
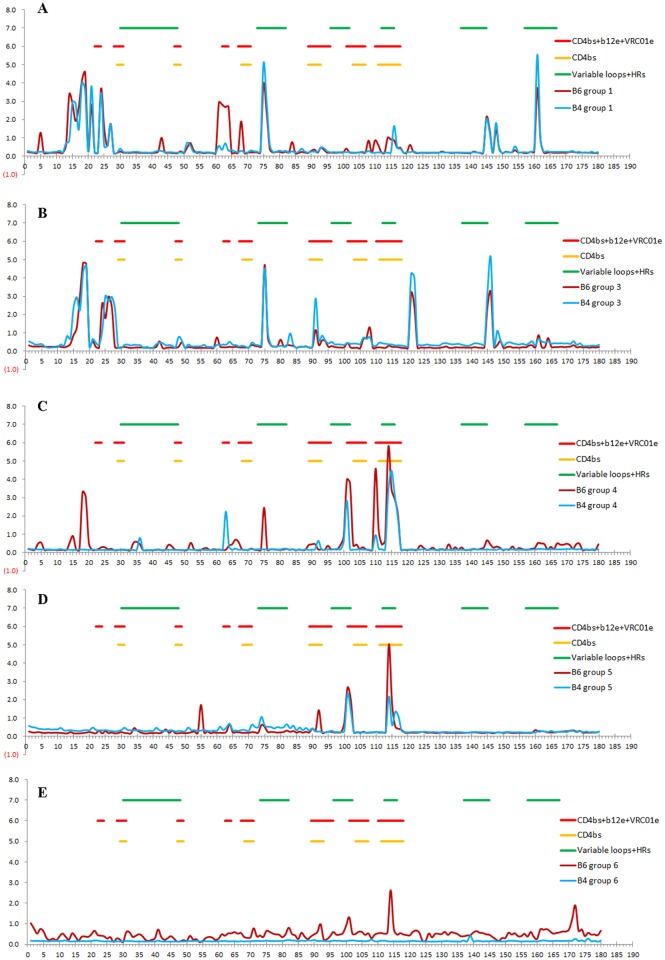
Profiling of bleed 4 and 6 rabbit IgGs from each group with the consensus clade B 15-mer peptides. Profiles of bleed 4 and 6 rabbit IgGs from group 1 (A), 3 (B), 4 (C), 5 (D) and 6 (E) with the consensus clade B 15-mer peptides are shown. Locations of CD4bs peptides, b12 epitope (b12e) and VRC01 epitope (VRC01e), as well as Env variable loops and HR regions (HR1 and HR2) are indicated according to “Neutralizing Antibody Resources” (http://www.hiv.lanl.gov/content/immunology/neutralizing_ab_resources.html). Two serum samples from a same group were profiled separately, but the addition results of the two samples from the same group are shown. X axis: position of the peptides. Y axis: OD450nm.

**Fig 4:**
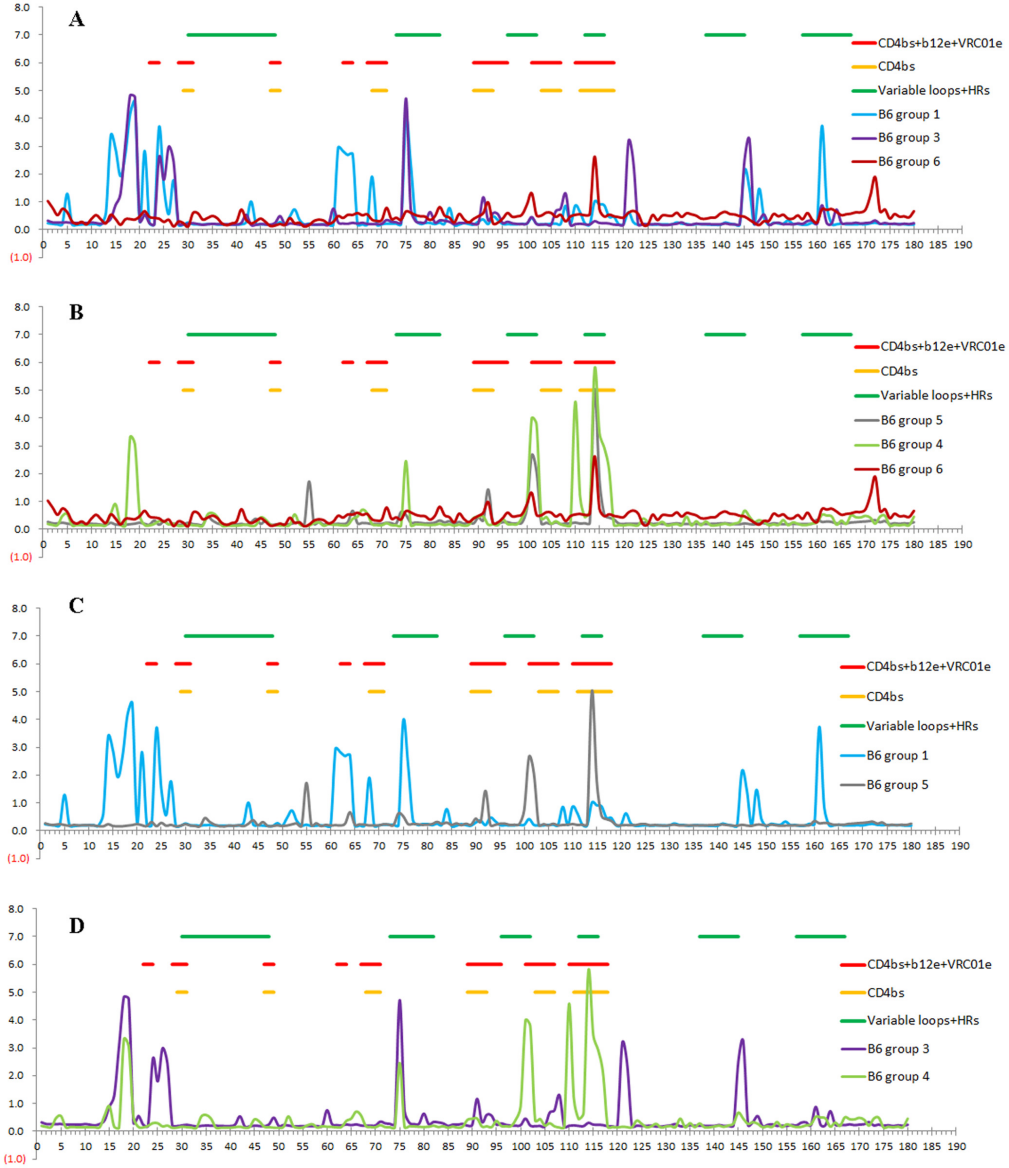
Comparison of profiles of different bleed 6 rabbit IgGs with consensus clade B 15-mer peptides. A-B: Profiles of rabbit IgGs from the immunization with or without (poly)peptide priming are compared. The profile of rabbit IgGs from immunization with P1-4 alone is included as a control. C: Profiles of rabbit IgGs from immunization with the same priming, but different order of the boosting immunogens are compared. D: Profiles of rabbit IgGs from two control groups without (poly)peptide priming and with different order of the boosting immunogens are compared. Locations of CD4bs peptides, b12 epitope (b12e) and VRC01 epitope (VRC01e), as well as Env variable loops and HR regions (HR1 and HR2) are indicated according to “Neutralizing Antibody Resources” (http://www.hiv.lanl.gov/content/immunology/neutralizing_ab_resources.html). Two serum samples from a same group were profiled separately, but the addition results of the two samples from the same group are shown. X axis: position of the peptides. Y axis: OD450nm.

To elucidate the mechanism underlying different immune responses resulting from different immunization protocols, we mapped bleed 4 and 6 IgGs with the whole panel of consensus clade B 15-mer peptides (Figs 3 and 4). Following priming with P1-4 and two boosts with gp140_SF162_, two additional boosts with RSC3 enhanced the elicitation of CD4bs antibodies (Fig 3A). In contrast, following the same priming and two boosts with RSC3, two additional boosts with gp140_SF162_ did not induce more diverse antibodies targeting CD4bs (Fig 3D). Multiple boosts with (poly)peptides were necessary to induce CD4bs Abs in the absence of Env boosting (Fig 3E). Immunization with P1-4 induced antibodies that targeted predominantly the CD4bs, and priming with (poly)peptides followed by boosts with gp140_SF162_ trimer and RSC3 enhanced the elicitation of CD4bs Abs and raised more diverse antibodies targeting different epitopes compared with immunization without (poly)peptide priming (Fig 4A). Interestingly, altering the order of the two boosting immunogens significantly affected the antibody profiles of the immunized rabbits. Following (poly)peptide priming, boosts with RSC3 and then gp140_SF162_ did not strongly enhance the elicitation of CD4bs Abs (Fig 4B), and the induced antibodies showed much less diversity in binding to consensus clade B peptides compared with boosts with gp140_SF162_ and then RSC3 (Fig 4C). This finding suggested that an initial boost with RSC3 might limit the diversity of B-cells that respond to the immunization. The two control groups with different orders of the two boosting immunogens also showed different antibody profiles (Fig 4D).

## DISCUSSION

Engineering the outer domain of Env gp120 to target putative VRC01 germline Ab has been reported [25]. The engineered outer domain can activate B-cells expressing putative germline VRC01 in vitro, but the immune response induced by the engineered outer domain in vivo remains unknown. Here, we tested four non-HIV (poly)peptides alone and in combination with gp140_SF162_ trimer and RSC3 in rabbits. We found that non-HIV (poly)peptides alone induced cross-reactive nAbs, and priming with the (poly)peptides followed by Env boosting enhanced eliciting cross-clade nAbs. We expressed (poly)peptide-Fc fusion proteins, but succeeded only with P3-, P4- and P6-Fc fusions. Recombinant P3-, P4- and P6-Fc fusions were used to prime group 2 rabbits followed by boosts with gp140_SF162_ trimer and then RSC3 (not shown). Although P3-, P4- and P6-Fc fusion proteins also showed priming effects compared to control groups 3 and 4 without priming, we did not include the data from group 2 rabbits herein for lack of comparability with the data from group 1, 5 and 6. We further found that properties of secondary immunogens and the order of the secondary immuogens for boosting significantly affected the outcome of the immunization. In this study, following non-HIV (poly)peptide priming, boosts with gp140_SF162_ trimer and then RSC3 (group 1) appears to work better than boosts with RSC3 and then gp140_SF162_ trimer (group 5) in inducing b12-competiting and cross-clade nAbs. The underlying mechanism remains to be elucidated. It seems that diversified initial immune responses might be important for subsequently focused immune responses to elicit cross-clade nAbs. Non-HIV immunogens that bind to putative germline bnAbs may help diversify the initial immune responses, which may initiate and guide the immune responses towards HIV-1 bnAbs upon stimulation with Envs.

We found that the degree of competition with mature b12 for binding to RSC3 did not correlate with the neutralization activity of rabbit IgGs (Fig 2D and 2E). Results from antibody profiling showed that (poly)peptide priming enhanced the elicitation of CD4bs Abs when the first boosting immunogen was gp140_SF162_ trimer. RSC3 enhanced the elicitation of CD4bs Abs when used following the (poly)peptide priming and trimer boosting, but it did not have the same effect when used immediately following (poly)peptide priming. These data are in agreement with the consensus view that immunogenicity is more complex than antigenicity.

The selected (poly)peptides bind to human and macaque b12 germline and iAbs, and P1 and P4 also bind to mature b12, but none of the (poly)peptides cross-reacts with VRC01 and 2F5, suggesting that they may specifically bind to putative b12 germline and iAbs. The five (poly)peptides used in rabbit immunization are constrained (poly)peptides. Although rabbit IgGs obtained from the immunization with the (poly)peptides alone bound to some linear peptides overlapping CD4bs (Fig 3E), the overall reading was low, thus, we cannot conclude that immunization with these (poly)peptides induced antibodies to linear peptides. Consensus clade B 15-mer peptides were used to map the rabbit IgGs herein. Some conformational epitopes that are recognized by the induced rabbit IgGs may have been missed in the mapping. We also cannot conclude that these peptides induced antibodies that bound to the same conformational epitopes recognized by mature b12. Some competing antibodies with mature b12 may be just binding antibodies, not b12-like neutralizing antibodies. These (poly)peptides may partially present b12 epitope, but they may initiate somatic mutations of germline b12 to such a degree that Env can bind and further stimulate the somatic maturation of b12 iAbs towards bnAbs.

According to IMGT, we synthesized putative rabbit b12 germline antibody heavy (IGHV1S8*01F, IGHD4-2*01 and IGHJ4*02F) and kappa light chain (IGKV1S19*01F and IGKJ1-3*01) variable genes and found that recombinant rabbit b12 germline antibody bound to the selected (poly)peptides in a dose-dependent manner (S3 Fig). Further study is required to investigate (poly)peptide-induced B-cell affinity maturation pathways in the rabbits. We have previously reported that one site mutation can convert non-binding human b12 germline antibody to Env-binding antibody; however, the introduction of neutralizing activity to human b12 germline antibody requires extensive somatic maturation [32]. Here, we used an extended 87-day immunization protocol. It remains to be investigated whether further prolongation of the immunization protocol can enhance elicitation of potent cross-clade nAbs [23].

The present study provides a proof-of-concept that non-HIV (poly)peptides might serve as primary immunogens to initiate immune responses towards elicitation of cross-clade neutralizing antibodies. Although priming with non-HIV (poly)peptides and boosting with gp140_SF162_ trimer and RSC3 elicited better neutralizing antibody responses than immunizing with gp140 and RSC3 in the absence of the non-HIV immunogens, the effect of non-HIV-immunogens was marginal and the responses overall were relatively weak; much weaker than mature b12. Other strategies for further improvement may be investigated in further studies, including multiple secondary immunogens, different boosting strategies and different adjuvants, etc. Non-HIV immunogens based on the putative germline antibodies of more potent bnAbs (e.g., VRC01, PG9, PGT128, PGT151, 10E8, etc) may also be identified to validate various strategies.

## Supporting Information

S1 Fig Binding of a panel of HIV-specific mAbs to recombinant gp140_SF162_ trimer (A) and RSC3 (B) by indirect ELISA. Two μg/mL of gp140_SF162_ or RSC3 were coated and 3-fold serially diluted mAbs with a starting concentration of 20 μg/mL added to the plates. Bound mAbs were detected by using HRP conjugated anti-human Fc (1:5,000) as secondary antibody and TMB as substrate. OD450nm was measured after color development at RT for 10min.
(TIF)

S2 Fig Titration of bleed 0, 2, 4 and 6 sera for SF162 gp140 trimer (A) and RSC3 (B) by indirect ELISA. Plates were coated with 2 μg/mL of SF162 gp140 or RSC3. Five-fold serially diluted rabbit sera were added to the plates. Bound rabbit IgGs were detected using HRP-conjugated anti-rabbit Fc as a secondary antibody and TMB as a substrate. The OD450nm was measured after color development at RT for 20 min.
(TIF)

S3 Fig Binding of putative rabbit b12 germline IgG antibody to the isolated (poly)peptides by ELISA. The plates were coated with 2 μg/mL of P1-4 and P6. Three-fold serially diluted rabbit b12 germline IgG1 were added to the plates. Bound rabbit b12 germline IgG1 were measured by HRP-conjugated anti-human Fc as a secondary antibody and TMB as a substrate. The OD450nm was measured after color development at RT for 20 min.
(TIF)
