# Supplementary figures and images for: Identification of Non-HIV Immunogens That Bind to Germline b12 Predecessors and Prime for Elicitation of Cross-clade Neutralizing HIV-1 Antibodies
Source: PLoS One. 2015 May 26;10(5):e0126428. doi: 10.1371/journal.pone.0126428 (PMC4444298; doi:10.1371/journal.pone.0126428)

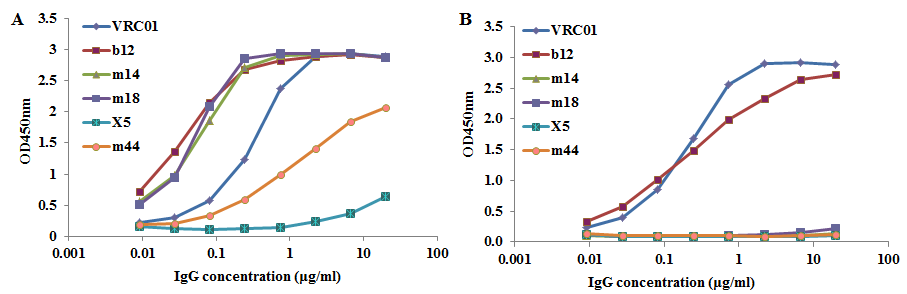

Supplement: S1 Fig — Two μg/mL of gp140SF162 or RSC3 were coated and 3-fold serially diluted mAbs with a starting concentration of 20 μg/mL added to the plates. Bound mAbs were detected by using HRP conjugated anti-human Fc (1:5,000) as secondary antibody and TMB as substrate. OD450nm was measured after color development at RT for 10min. (TIF) [file pone.0126428.s001.tif]

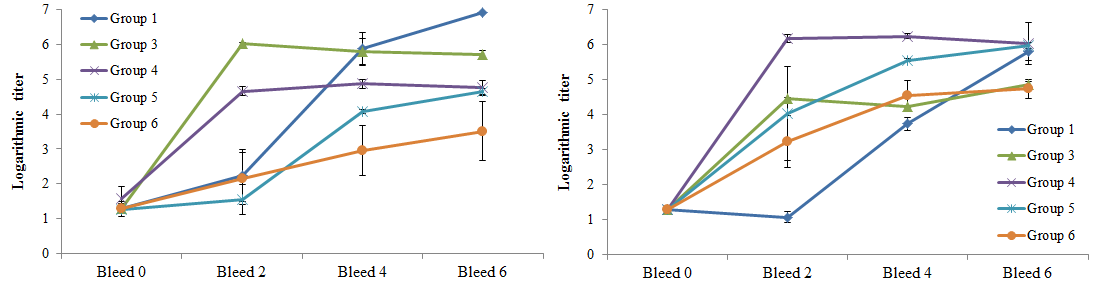

Supplement: S2 Fig — Plates were coated with 2 μg/mL of SF162 gp140 or RSC3. Five-fold serially diluted rabbit sera were added to the plates. Bound rabbit IgGs were detected using HRP-conjugated anti-rabbit Fc as a secondary antibody and TMB as a substrate. The OD450nm was measured after color development at RT for 20 min. (TIF) [file pone.0126428.s002.tif]

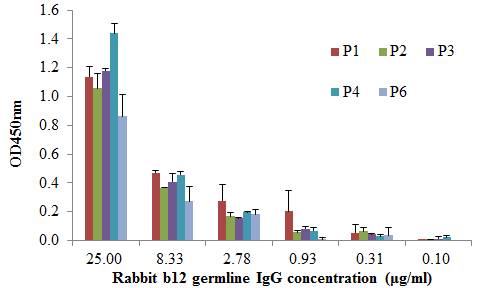

Supplement: S3 Fig — The plates were coated with 2 μg/mL of P1-4 and P6. Three-fold serially diluted rabbit b12 germline IgG1 were added to the plates. Bound rabbit b12 germline IgG1 were measured by HRP-conjugated anti-human Fc as a secondary antibody and TMB as a substrate. The OD450nm was measured after color development at RT for 20 min. (TIF) [file pone.0126428.s003.tif]
